# Interactions of Indole Derivatives with β-Cyclodextrin: A Quantitative Structure-Property Relationship Study

**DOI:** 10.1371/journal.pone.0154339

**Published:** 2016-04-28

**Authors:** Milan Šoškić, Ivana Porobić

**Affiliations:** Department of Chemistry, Faculty of Agriculture, University of Zagreb, Svetošimunska 25, 10000 Zagreb, Croatia; University of Parma, ITALY

## Abstract

Retention factors for 31 indole derivatives, most of them with auxin activity, were determined by high-performance liquid chromatography, using bonded β-cyclodextrin as a stationary phase. A three-parameter QSPR (quantitative structure-property relationship) model, based on physico-chemical and structural descriptors was derived, which accounted for about 98% variations in the retention factors. The model suggests that the indole nucleus occupies the relatively apolar cavity of β-cyclodextrin while the carboxyl group of the indole -3-carboxylic acids makes hydrogen bonds with the hydroxyl groups of β-cyclodextrin. The length and flexibility of the side chain containing carboxyl group strongly affect the binding of these compounds to β-cyclodextrin. Non-acidic derivatives, unlike the indole-3-carboxylic acids, are poorly retained on the column. A reasonably well correlation was found between the retention factors of the indole-3-acetic acids and their relative binding affinities for human serum albumin, a carrier protein in the blood plasma. A less satisfactory correlation was obtained when the retention factors of the indole derivatives were compared with their affinities for auxin-binding protein 1, a plant auxin receptor.

## Introduction

Cyclodextrins [1] are cyclic oligosaccharides of toroidal shape, made up of six (α-cyclodextrin), seven (β-cyclodextrin), eight (γ-cyclodextrin) or more glucose residues linked by α-1,4-glycosidic bonds. The central cavity of cyclodextrins, which is lined with methylene hydrogens and glycosidic oxygen bridges, is relatively hydrophobic compared to water. On the other hand, the primary and secondary hydroxyl groups, located on the smaller and larger openings of the cyclodextrin torus, respectively, make their outer surface polar. Cyclodextrins have ability to make inclusion complexes with various organic molecules of appropriate size, shape and polarity, which is widely exploited in pharmaceutical industry, food technology, environmental protection and analytical chemistry [2]. Cyclodextrins also found applications as model systems for studying enzyme-substrate interactions [3]. However, the mechanism of complex formation between cyclodextrins and guest molecules is still not fully elucidated.

Indole-3-acetic acid is the most important auxin in plants, which controls nearly all aspects of plant growth and development [4]. In a search for auxin receptor(s) a number of proteins with ability to bind auxins have been detected [5]. It was only recently that transport inhibitor response 1 protein (TIR 1) [6, 7] and auxin-binding protein 1 (ABP 1) [8] have been identified as the long-term sought auxin receptors. Whereas TIR1-mediated auxin signaling pathway is rather well characterized [9], the mode of action of ABP 1 remains unclear.

IAA and its ring-substituted derivatives also attracted attention as potential pro-drugs for use in targeted cancer therapy as it was observed that the oxidation products of these compounds produced by horseradish peroxidase are toxic to tumor cells *in vitro* [10, 11].

**Fig 1:**
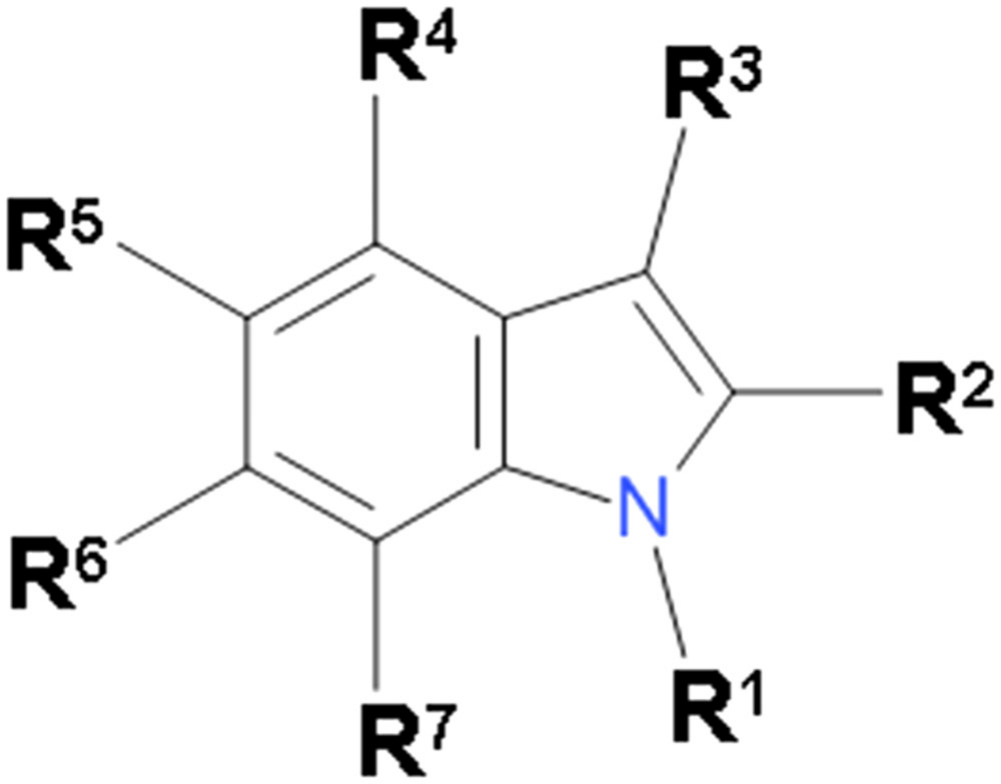
General structure of indole derivatives.

The interactions of β-cyclodextrin with the indole derivatives which possess auxin activity have been subject of several studies [12–14]. However, in all these studies only a small number of the indole derivatives were tested. Here we report the retention factors for 31 indole derivatives (Fig 1), obtained by high-performance liquid chromatography, using immobilized β-cyclodextrin as a stationary phase in the reversed-phase mode. In this mode, retention factors are mostly determined by the stability of inclusion complexes formed between a solute and the stationary phase. In order to get insight into physico-chemical and structural factors affecting the stability of inclusion complexes between the indole derivatives and β-cyclodextrin, a QSPR (quantitative structure-property relationship) analysis was performed. We also correlated the retention factors of the indole derivatives with their binding affinities for ABP1 [15, 16] and immobilized human serum albumin [17] to explore to what extent the retention mechanism of the cyclodextrin bonded phase parallels the mechanism of auxins recognition by the plant receptor or by the aforementioned carrier protein in the blood plasma.

## Materials and Methods

### Experimental

#### HPLC Instrument

High-performance liquid chromatography was performed using Varian 920-LC (Agilent Technologies, Santa Clara, United States) equipped with a low pressure quaternary pump with built in four channel Degasser^™^, a photodiode array detector, a column heater and an auto sampler with 100 μL sample loop. The system is controlled by Varian Galaxie^™^ Chromatography Software.

#### Determination of Retention Factors

Chromatography was performed on an Astec Cyclobond^™^ I 2000 column, packed with β-cyclodextrin bonded to a silica gel support (150 x 4.6 mm id; Sigma-Aldrich/Supelco, Bellefonte, USA), using a mixture of 0,1% triethylamine acetate (TEAA) buffer (pH 4,1) and acetonitrile (80:20 v/v) as a mobile phase.

Retention times (*t*_r_) of indolic compounds were determined independently, injecting 20 μL of the stock solution (~1mg/mL). Flow rate was 1.0 mL/min. The column temperature was maintained at approximately 29°C. The retention time was average of at least two measurements. The dead time (*t*_0_) was determined from the retention time of acetone which is not retained by the column. The chromatographic retention of the solutes was expressed as the retention factor (*k*):

$$k=\frac{t_{r}-t_{0}}{t_{0}}$$ (1)

All chemicals used were of analytical grade or better.

### Data Analysis

#### Molecular Descriptors

The molecular descriptors used in this study were either extracted from a standard compilation [18] or were calculated by the commercial software TSAR 3.3 for Windows [19] and Bio-Loom for Windows [20], with the exception of experimental log *P* values for compounds 1, 5–18, 20 and 28 (Table 1), which were taken from reference 17.

**Table 1:** Octanol-water partition coefficient and indicator variables (meaning given in Results and discussion) plus observed and calculated retention factors of the studied indole derivatives obtained on Astec CyclobondTM I 2000 column.

No.	Compound	log *P*	*I*_1_	*I*_2_	log *k*_CD_	
					Exp.	Eq 9
1.	Indole-3-aceticacid(IAA)	1.42	0	2	0.65	0.62
2.	Indole-3-carboxylicacid	2.13	0	1	0.13	0.21
3.	Indole-3-acrylicacid	2.23	1	2	0.54	0.51
4.	Indole-3-butyricacid	2.27	0	2	0.78	0.76
5.	2-Me,5-OMe-IAA	2.14	1	2	0.46	0.49
6.	4-F-IAA	1.66	0	2	0.57	0.66
7.	4-Cl-IAA	2.13	0	2	0.60	0.74
8.	5-F-IAA	1.72	0	2	0.68	0.67
9.	5-Cl-IAA	2.38	0	2	0.88	0.78
10.	5-Br-IAA	2.57	0	2	0.94	0.81
11.	5-Me-IAA	1.99	0	2	0.64	0.71
12.	5-OMe-IAA	1.23	0	2	0.58	0.58
13.	5-OBz-IAA	3.20	0	2	1.04	0.92
14.	5,7-Cl_2_-IAA	3.42	0	2	0.86	0.96
15.	6-F-IAA	1.74	0	2	0.68	0.67
16.	6-Me-IAA	1.96	0	2	0.72	0.71
17.	7-F-IAA	1.81	0	2	0.71	0.68
18.	7-Cl-IAA	2.32	0	2	0.81	0.77
19.	7-Br-IAA	2.44	0	2	0.85	0.79
20.	7-Me-IAA	1.95	0	2	0.61	0.71
21.	Et-ester-IAA	2.34	0	0	-0.33	-0.28
22.	Indole-3-acetamide	0.44	0	0	-0.58	-0.62
23.	Indole-3-ethanol	1.32	0	0	-0.33	-0.45
24.	Indole-3-methylketone	1.98	0	0	-0.39	-0.32
25.	Indole-3-ol-acetateester	1.62	0	0	-0.40	-0.40
26.	Indole-3-acetonitrile	1.55	0	0	-0.97	-0.41
27.	1-Me-IAA	1.87	0	2	0.64	0.69
28.	6-Cl-IAA	2.41	0	2	0.93	0.79
29.	Indole-3-propionicacid	1.89	0	2	0.65	0.70
30.	Me-ester-IAA	1.81	0	0	-0.37	-0.37
31.	Indole-3-aldehyde	1.95	0	0	-0.46	0.34

#### Model Generation and Validation

The data set was randomly divided into a training set (about 80% compounds of the whole data set) and a validation set (about 20% compounds of the whole data set) using a training/validation set splitting routine available in MobyDigs software [21]. The training set was used for generation of the QSPR models. The most appropriate descriptors for the QSPR modeling were selected by the stepwise multiple linear regression technique [22] as implemented in TSAR 3.3. In the stepwise regression the first descriptor which enters the model is one with the highest correlation coefficient with response variable. New descriptors are then added one at a time and their importance for the model is checked by *F*-statistics. If the *F* value of a descriptor falls below a prespecified value the variable is removed. At each step, before a new variable is added, it is checked if the descriptors already included in the model can be removed. The procedure terminates when no more variables can be added or removed from the model according to the prespecified *F*-to-enter and *F*-to-remove values. In our case *F*-to-enter and F-to-remove values were set to 4.

In all regression equations *n* is the number of compounds used in the analysis, *r*^2^ is the squared correlation coefficient, *s* is the standard deviation of the estimates, and *F* is the ratio of the variance accounted for regression and the residual variance. The degrees of freedom (*k*, *n-k*-1) associated with *F* are specified in the superscript, wherein *k* is the number of independent variables in the equation.

The robustness and predictive ability of the models were evaluated by internal and external validation techniques [23–25] using MobyDigs software. The models were internally validated by leave-one-out and response randomization (*Y*-scrambling) procedure. The validation parameters considered in the leave-one-out procedure were *q*^2^_LOO_ (the cross-validated coefficient of determination) and *PRESS* (the sum of squared prediction errors from the leave-one-out cross-validation analysis).

In the response randomization procedure, the *Y* data of the original model are randomized and a new regression model is generated using intact *X* data. The procedure is usually repeated several hundred times. It is expected that the *r*^*2*^_Yscrambling_ and *q*^*2*^_Yscrambling_ values of the thus obtained models are, in general, significantly lower than those of the original one, fitted to the unscrambled *Y* data. The results of the randomization procedure can be summarized [26] by calculating *Y*-scrambling parameters a(*r*^2^_Yscrambling_) and a(*q*^2^_Yscrambling_) which are the intercepts of the equations:

$$r_{Yscrambling}^{2}=a+br$$ (2)

$$q_{Yscrambling}^{2}=a+br$$ (3)

In the above equations *r*^*2*^_Yscrambling_ and *q*^*2*^_Yscrambling_ are the squared correlation coefficient and the cross-validated coefficient of determinations of the models obtained using the same predictors but *Y*-scrambled data and *r* is the correlation coefficient between the *Y* data of the original model and *Y*-scrambled data. If the *Y*-scrambling parameter values of the thus obtained models satisfy the following criteria,

$$a\left(r_{Yscrambling}^{2}\right)<0.3$$ (4)

$$a\left(q_{Yscrambling}^{2}\right)<0.05$$ (5)

then the risk of chance correlation for the original model is negligible.

External validation of the final model was performed on a validation set of five compounds not used in the generation of the model. The external predictive capability of the model was quantified by the predictive squared correlation coefficients *q*^2^_EXT_ and the external standard deviation error of prediction *SDEP*_EXT_.

The applicability domain of the final model was assessed and visualized by the Williams plot, a scatter plot of the standardized cross-validated residuals versus leverages (or hat values) h, which are defined as follows,

$$h_{i}=x_{i}^{T}{\left(X^{T}X\right)}^{-1}x_{i}$$ (6)

where *x*_i_ is the descriptor row vector of the considered compound and *X* is the descriptor matrix derived from descriptor values of the training set. This graph allows detecting outliers and defines the boundary of the applicability domain. Compounds with standardized residuals greater than 3 are considered to be outliers, while influential compounds are those with a leverage (*h*) higher than the critical value h* (h* = 3*p*′/*n*, where *p*′ is the number of predictor variables plus one, and *n* is the number of training compounds. Predictions for compounds whose leverage values exceed the critical one should be considered unreliable.

## Results and Discussion

Experimental and calculated retention factors (expressed as log *k*_CD_) for 31 indole derivatives (Fig 1), most of them with auxin activity, are listed in Table 1, together with corresponding physico-chemical and structural descriptors which appear in the final model. The data set consists of 23 indole carboxylic acids, including three out of four naturally occurring auxins (IAA, 4-Cl-IAA and indole-3-butyric acid) [27] plus 8 non-acidic indole derivatives. The latter are included in the data set to assess the contribution of the carboxyl group to the stability of inclusion complexes. Compounds 1–26 were used as a training set, while compounds 27–31 were used as a validation set. The following descriptors have been used in the screening process: octanol-water partition coefficient (log *P*), molar refractivity (*MR*), molar volume (*MgVol*), dipole moment (*μ*), hydrophobic (*π*) and Verloop’s steric constants (*L*, *B*_1_, *B*_5_) for substituent position 4–7, Hammett’s electronics sigma constants (*σ*_m_, *σ*_p_) for substituent positions 4–7, number of hydrogen bond donors (*H*d), number of hydrogen bond acceptors (*H*a), and two indicator variables, whose meaning will be given later.

Initially a QSAR model for IAA and its ring-substituted derivatives (compounds 1, 6–13, and 15–20) was developed:

$$logk_{CD}=0.219\left(\pm 0.173\right)+0.252\left(\pm 0.082\right)logP$$ (7)

$$n=15\;\;\;\;r^{2}=0.769\;\;\;\;s=0.071\;\;\;\;F^{1,13}=43.2\;\;q^{2}=0.711\;\;\;\;PRESS=0.082$$

(For their atypical chromatographic behavior 2-Me, 5-OMe-IAA and 5,7-Cl_2_-IAA were excluded from the analysis at this phase of the model development.) In this and the following equation 95% confident intervals were given in parentheses. The significance of all the derived models is above the 95% level or higher. It can be seen from the model that more lipophilic indole-3-acetic acids are generally longer retained on the column, which confirms the claim that inclusion complexes are formed between a solute and immobilized β-cyclodextrin during the chromatographic process. In order to generate a model which describes the chromatographic behavior for all compounds in the training set two indicator variables (*I*_1_ and *I*_2_) had to be introduced in the model.

$$logk_{CD}=-0.769\left(\pm 0.193\right)+0.172\left(\pm 0.101\right)logP-0.250\left(\pm 0.206\right)I_{1}\;+0.571\left(\pm 0.071\right)I_{2}$$ (8)

$$n=26\;\;\;\;r^{2}=0.948\;\;\;\;s=0.133\;\;\;\;F^{3,22}=134.6\;\;q^{2}=0.926\;\;\;\;PRESS=0.558$$

A model with much better statistics can be obtained by omitting indole-3-acetonitrile, a response outlier (Williams plot not shown), from regression analysis:

$$logk_{CD}=-0.678\left(\pm 0.117\right)+0.171\left(\pm 0.060\right)logP-0.248\left(\pm 0.121\right)I_{1}+0.526\left(\pm 0.044\right)I_{2}$$ (9)

$$n=25\;\;\;\;r^{2}=0.977\;\;\;\;s=0.078\;\;\;F^{3,21}=295.1\;\;q^{2}=0.968\;\;\;PRESS=0.178$$

Indicator variable *I*_1_ amounts 1 for 2-Me, 5-OMe-IAA and indole-3-acrylic acid and 0 for the rest of indole derivatives. Namely, the experimentally determined retention factors for 2-Me, 5-OMe-IAA and indole-3-acrylic acid are lower than those predicted from their log *P* values Eq (7). The reasons for this are probably steric in nature. In addition to hydrophobic interactions, as it will be elaborated later, the inclusion complexes are also stabilized by hydrogen bonding between β-cyclodextrin and the indole derivatives. A conformational study by Antolić et al [28] revealed that 2-methylindole-3-acetic acid has different conformational preferences than IAA, due to the presence of methyl group in *ortho* position, which could preclude optimal interactions between the carboxyl group of the respective derivative and the hydroxyl groups on the openings of the torus. Similar negative steric influences on the retention times have been observed in our previous studies [17, 29, 30]. In case of indole-3-acrylic acid the optimal interactions might be hindered by the reduced flexibility of its 3-side chain caused by the presence of a double bond. Unsaturated compounds have lower conformational degrees of freedom than the corresponding saturated compounds. The fact that indole-3-butyric acid, having a similar lipophilicity as indole-3-acrylic acid, but a more flexible 3-side chain, is retained longer on the column than indole-3-acrylic acid strongly supports above statement. Since the aforementioned negative steric effects are comparable in magnitude, they are covered in the model by a common indicator variable *I*_1_. It should be also noted that the retention factor for 5,7-Cl_2_-IAA is comparable to those for the mono halogenated analogues (i.e. 5-Cl-IAA and 7-Cl-IAA). Obviously the presence of additional lipophilic substituent in the structure did not lead to additional stabilization of the complex between 5,7-Cl_2_-IAA and β-cyclodextrin. A possible rationalization for this discrepancy can be that the substituted indole ring, presumably for steric reasons, is not completely embedded in the β-cyclodextrin cavity.

Indicator variable *I*_2_ shows the importance of the carboxyl group for the inclusion complexes formation. It was assigned a value of 2 for compounds with the 3-side chain containing carboxyl group, 1 for the derivatives with carboxyl group directly attached to indole nucleus at substituent position 3 and 0 for the non-acidic indole derivatives. Clearly, the carboxyl group is more effective in forming polar interactions with β-cyclodextrin than corresponding functional groups of the non-acidic indole derivatives. Namely, as can be seen from Table 1, log *P* values of the non-acidic indole compounds (with the exception of indole-3-acetamide) are similar to that of IAA or higher. The poor retentions of the non-acidic derivatives clearly demonstrate that the aromatic moiety alone, without an adequate polar group, is not sufficient for the formation of stable inclusion complexes. In other words, the complexation of the indolic compounds with β-cyclodextrin is a result of a cooperative effect of hydrophobic interactions and hydrogen bonding. On the other hand, the low retention factor of indole-3-carboxylic acid indicates that the compatibility of interacting apolar and polar areas of a solute and β-cyclodextrin may be of critical importance for the formation of strong inclusion complexes. This is supported by the fact that increases in the length of the alkyl chain containing the carboxyl group results in more stable inclusion complexes.

It is not clear whether the indole NH group participates in hydrogen bonding with the stationary phase, regarding that there is practically no difference in the retention times between IAA and its 1-methyl analogue (compound 27).

**Fig 2:**
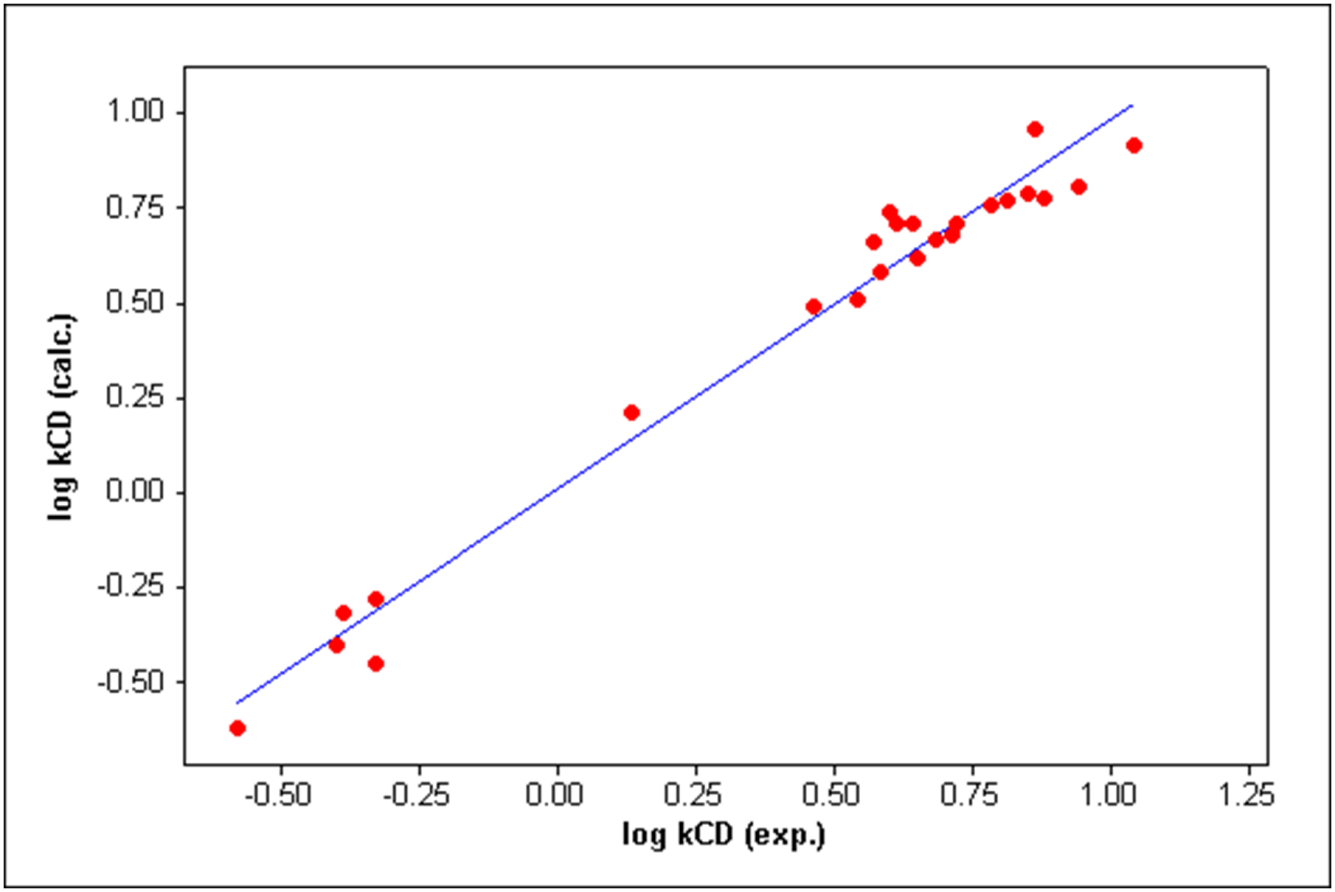
The observed versus calculated log *k*_CD_ values for the studied indole derivatives Eq (9).

The statistical parameters of Eq 9 show that the model is robust. A plot of the observed retention factors of the indole derivatives versus the log *k*_CD_ values calculated by Eq 9 is given in Fig 2. The correlation matrix (Table 2) shows that the collinearity between the predictor variables in Eq 9 is negligible.

**Table 2:** Correlation matrix for variables used in Eq 9.

	log *P*	*I*_1_	*I*_2_
log *P*	1		
*I*_1_	0.08	1	
*I*_2_	0.39	0.16	1

The probability of chance correlation for the final model Eq (9) was tested using the *Y*-scrambling procedure, which was repeated 300 times. It was found that *Y*-scrambling parameters of the model (a(*r*^2^_Yscrambling_) = 0.06; a(*q*^2^_Yscrambling_) = -0.38) are below the threshold values (a(*r*^2^_Yscrambling_) < 0.3; a(*q*^2^_Yscrambling_) < 0.05), which strongly suggest that the model is not a result of chance correlation.

The quality of the model was further checked by external validation using validation set of five compounds (compounds 27–31 in Table 1). The high value of *q*^2^_EXT_ (0.98) and a low value of *SDEP*_EXT_ (0.088) obtained in this test confirmed the robustness of the model.

**Fig 3:**
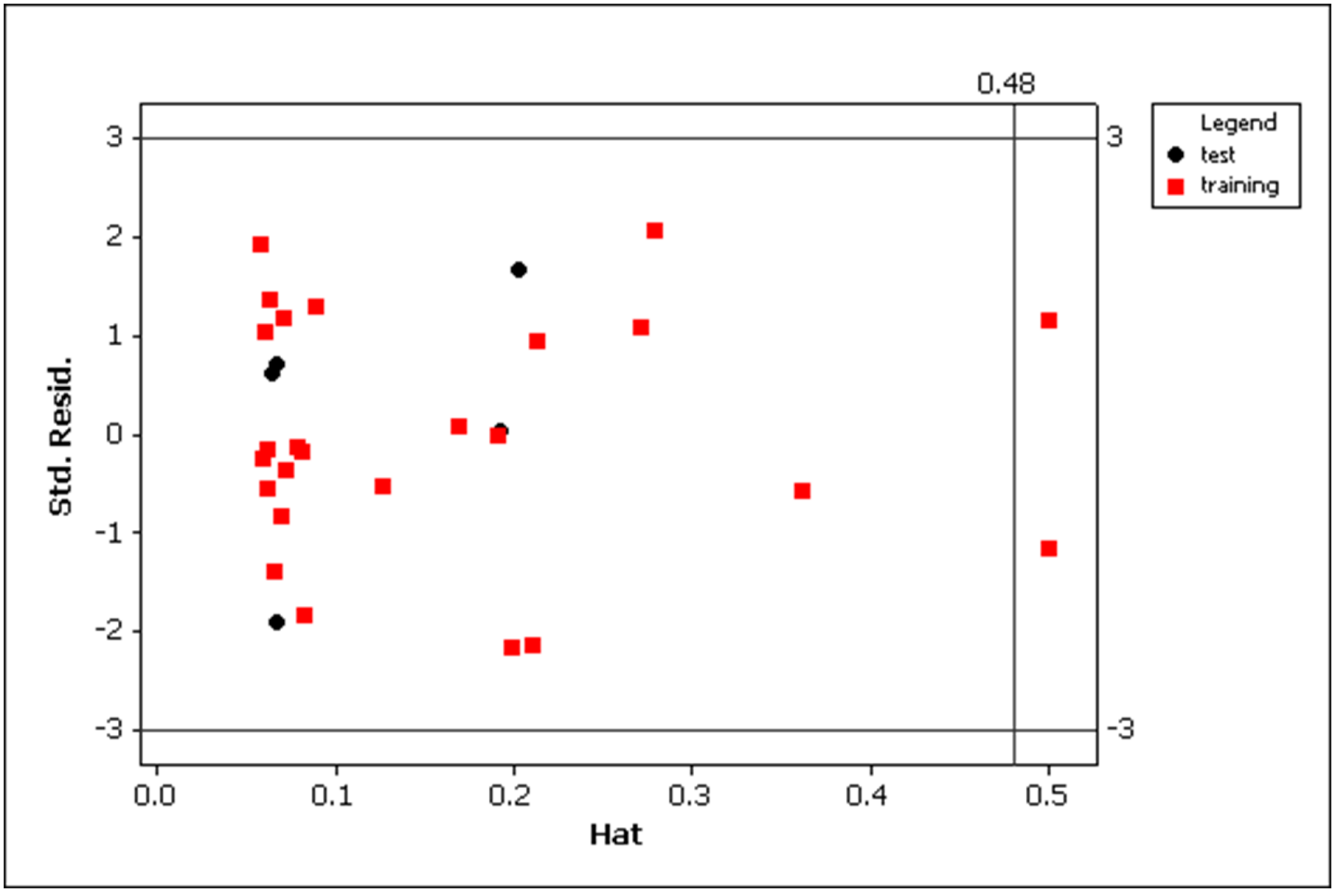
Williams plot of standardized residuals (*y*-axis) versus hat values (*x*-axis) for the final model Eq (9). The horizontal and vertical lines represent ± 3 standardized residuals and warning hat value (h* = 0.48), respectively. The compounds 3 and 5 are slightly influential compounds in the training set, with hat values (h = 0.50) somewhat higher than the warning hat value.

The Williams plot (Fig 3), for the final model Eq (9), reveals that compounds 3 and 5 (Table 1) are slightly influential compounds in the training set, with leverage values somewhat higher than the warning leverage (h* = 0.48).

**Fig 4:**
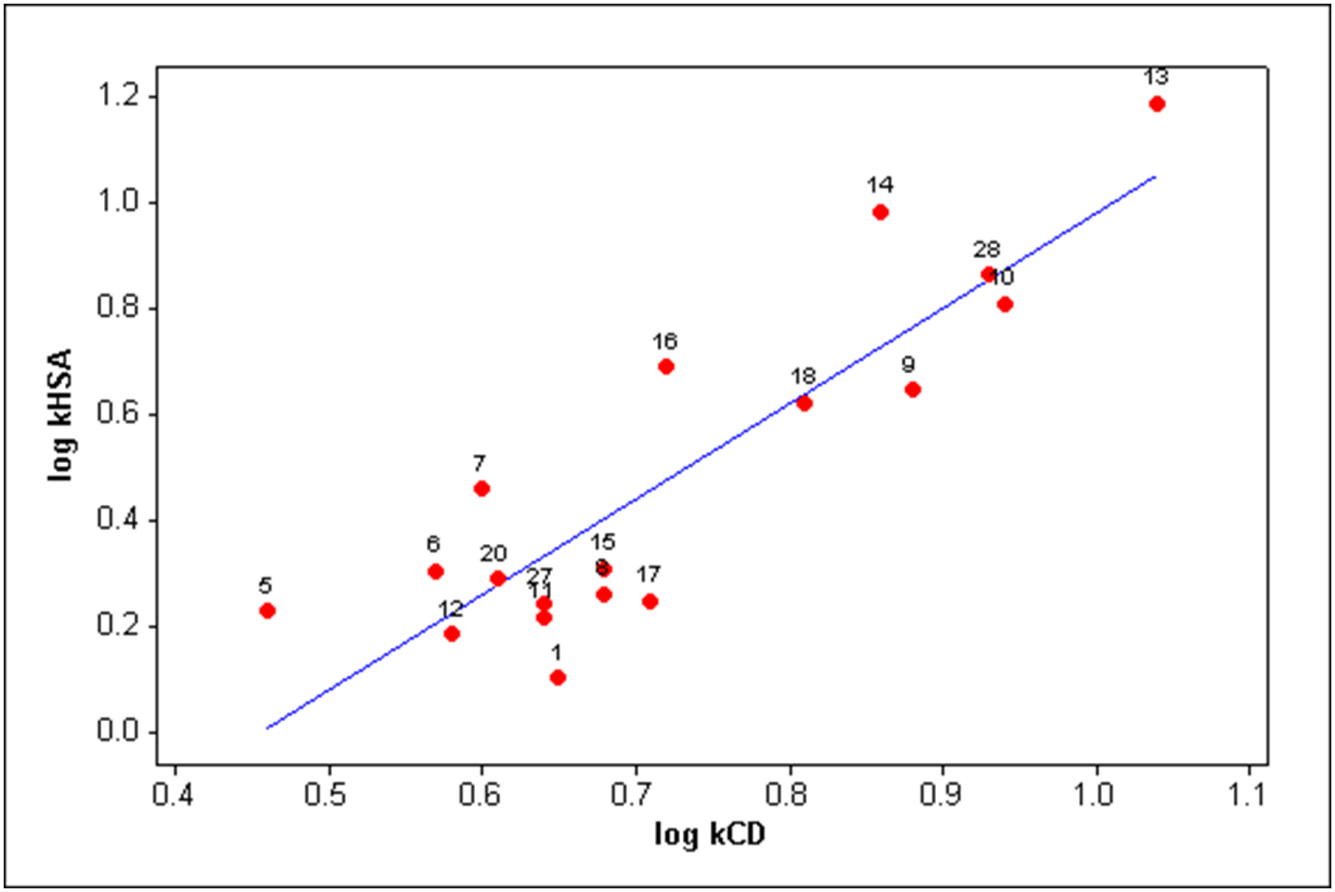
Scatter plot of the relationship between relative binding affinities of indole derivatives for human serum albumin (log *k*_HSA_) and their retention factors obtained on Astec Cyclobond^™^ I 2000 column (log *k*_CD_). The equation for the regression line is: log *k*_HSA_ = -0.82 (±0.40) + 1.80 (±0.53) log *k*_CD_; *n* = 18, *r*^2^ = 0.76, *s* = 0.16, *F*^1,16^ = 52.2.

In this section we will compare the retention factors of indole derivatives with their (relative) binding affinities for auxin-binding protein 1 [15, 16] (Table 3) and immobilized human serum albumin [17] (HSA, Fig 4). Human serum albumin is a soluble protein which is involved in the transportation of many low-molecular-weight compounds in blood. The binding affinities of drugs to this protein affect their absorption, distribution, metabolism and excretion, and thus their therapeutic effect. An X-ray crystallographic study of interactions between napthalene-1-acetic acid (a synthetic auxin) and ABP1 have been reported in 2002 [31]. It was found that mostly lipophilic binding cavity of the protein accommodates the aromatic nucleus of naphthalene-1-acetic acid while its carboxylate group interacts with Zn^+2^ ion, coordinated by three histidine residues and a glutamate. Similarly, the indole-benzodiazepine binding site on HSA, which is specific for small aromatic carboxylic acids, was found to be a deep lipophilic pocket with positively charged entrance [32]. The fact that β-cyclodextrin shares a similar binding motif with the aforementioned proteins prompt us to examine whether there exist any correlation between the retention factors of the indole derivatives and their affinities for ABP1 and HSA. Although the molecular composition of β-cyclodextrin is quite different from those of the binding sites of ABP1 and HSA, β-cyclodextrin may respond to structural variations of the indole derivatives in a fashion similar to the native receptors. It worth noting that the affinities for ABP1 were experimentally determined for only several indole derivatives [15, 16]. It should be also pointed out that a reasonably well correlation was found between stability constants for inclusion complexes of β-cyclodextrin with various organic solutes and their retention times observed on the immobilized cyclodextrin [33].

**Table 3:** Retention factors (*log k*_CD_) obtained on Astec Cyclobond^™^ I 2000 column plus experimental and calculated binding constants (p*K*_d_) to ABP1 for nine indole derivatives. The *pK*_d_ (*calc*.) values were calculated from the equation: p*K*_d_ = 3.82 (±0.50) + 1.91 (±0.87) log *k*_CD_; *n* = 8, *r*^2^ = 0.83, *s* = 0.52, F^1,6^ = 28.5. Compound 9 was excluded from the regression.

No.	Compound	*log k*_CD_	p*K*_d_ (*exp*.)	*pK*_d_ (*calc*.)
1.	Indole-3-acetic acid	0.65	5.4^a^	5.06
2.	Indole-3-propionic acid	0.65	5.2^a^	5.06
3.	Indole-3-butyric acid	0.78	5.0^a^	5.31
4.	4-chloroindole-3-acetic acid	0.60	5.6^b^	4.96
5.	Indole-3-acrylic acid	0.54	4.0^a^	4.85
6.	Indole-3-ol-acetate ester	-0.40	3.5^a^	3.05
7.	Indole-3-methyl ketone	-0.39	2.9^a^	3.07
8.	Indole-3-aldehyde	-0.46	2.7^a^	2.94
9.	Indole-3-acetonitrile	-0.97	4.0^a^	1.97

^a^ taken from reference [15]

^b^ taken from reference [16]

A series of structure-activity relationship studies have been carried out [34–40] in order to gain a deeper understanding of growth promoting effects of auxins. A particularly interesting paper for our work is a study by Edgerton et al. [41], in which they correlate structural features with binding affinities of different auxin like molecules for ABP1, with goal to determine their likely bound conformations. The study included, among other auxin analogues, the indole derivatives (Table 3), whose binding affinities for ABP1 we will compare with their retention factors. Mostly because of anomalous chromatographic behavior of indole-3-acetonitrile a low correlation was obtained between the binding affinities and retention factors of the indolic compounds. Omitting this compound improved the correlation markedly (*r*^2^ = 0.83). In order to find out what lies behind the above correlation we attempted to construct a simple quantitative model which could explain most of the variations in the p*K*_d_ values of the indole derivatives. It was found that an indicator variable which differentiate indole-3-acidic acids (I = 1) from nonacidic analogues (I = 0) explained practically as much variation in the p*K*_*d*_ values as chromatographic parameter log *k*_CD_ (80%). The closer inspection of Tables 1 and 3 shows that the result is not unexpected. Similarly as in case of the retention factors, the affinities of indole-3-carboxylic acids are in general significantly higher than those of the non-acidic analogues. Since structural differences of the majority of compounds displayed in Table 3 are related to the 3-side chain, variations in their binding affinities mostly reflect the differences in the binding capacities of their polar groups to the receptor site. A recent molecular dynamic simulation study [42] has shown that the interaction between the carboxyl group of IAA and the zinc ion of the binding site was very strong. It seems reasonable to assume that the rest of the indole-3-carboxylic acids also form favorable interactions with the Zn ion. The somewhat lower affinity of indole-3-acrylic acid could be rationalized by its restricted flexibility. On the other hand, the lower binding affinities of the non-acidic derivatives may be caused by either the diminished ability of their polar functional groups to bind to zinc ion or by a different binding mode of these compounds. It should be noted that in previously mentioned paper [41] (published before the structure of auxin binding place of ABP1 was resolved), Edgerton et al rationalized the low affinities of indole-3-aldehyde and indole-3-methyl ketone by the assumption that their polar functional groups do not interact with a binding element for the carboxylate group. Hence the correlation between p*K*_*d*_ values and retention factors of the indole compounds in Table 3 seems to be consequence of the fact that hydrogen bonding capacity of indole-3-carboxylic acids is higher than those of the non-acidic indole derivatives and that they are also generally better zinc binding groups compared to the latter.

In contrast to the compounds considered previously, variations in the relative binding affinities of indole-3-acetic acids for HSA are mostly result of the interactions between the substituted indole rings and amino acid residues within the lipophilic pocket. Namely, interactions between the carboxylate group and positively charged amino acid residues at the entrance of the binding sites are not likely to be significantly influenced by the substituent groups on the ring, because of buffering effect of a methylene group separating the carboxylate group and the indole nucleus. A fairly good correlation (*r*^2^ = 0.765, Fig 4) was obtained when compared the retention factors of indole-3-acetic acids (compounds 1, 5–18, 20, 27 and 28 in Table 1) with their relative binding affinities for the immobilized human serum albumin [17]. Comparison of Eq 9 with the original model [17] describing the affinities of ring-substituted indole-3-acetic acids for HSA (log *k*_HSA_ = - 0.463 + 0.398 log *P* + 0.244 *E*_S_ (R^2^) - 0.339 *E*_S_ (R^6^) + 0.448 σ (R^5^); *n* = 30, *r*^2^ = 0.966, *s* = 0.080, *F*^4,25^ = 175.6) shows that lipophilicity plays an essential role in the binding of the indole derivatives to both β-cyclodextrin and HSA, or to put it another way, this molecular property contributes most to the above determined correlation. The correlation also implies that the binding site of HSA is not sterically more demanding than β-cyclodextrin. In fact it is more spacious than the apolar cavity of β-cyclodextrin. The binding pocket is about 16 Å deep and about 8 Å wide [43], whereas the internal diameter of β-cyclodextrin is approximately 5.6 Å and the height is 7.8 Å [14]. On the other hand, the presence of steric (*E*_S_ (R^6^)) and electronic (σ (R^5^)) terms in the above model points to the specific interactions which have not been observed in the complexation process between the indole derivatives and β-cyclodextrin.

## Conclusion

The retention factors for 31 indole derivatives, obtained by high-performance liquid chromatography on a β-cyclodextrin stationary phase, were correlated with series of physico-chemical and structural descriptors. A very good three-parameter QSPR model was obtained which accounts for about 98% variations in the retention factors. The model confirms that the retention mechanism is based on the formation of inclusion complexes between indole derivatives and the stationary phase. The stability of the complexes strongly depends on a cooperative effect of hydrophobic interactions and hydrogen bonding between indole derivatives and immobilized β-cyclodextrin. As a rule, lengthening of the 3-side chain favors the binding of indole-3-carboxylic acids to the stationary phase. Anomalous retention behavior of indole-3-carboxylic acid suggests that the compatibility between interacting hydrophilic and hydrophobic areas of the indole derivatives and β-cyclodextrin is another factor of critical importance for the strong inclusion complexation. The lower retention factors of the 2-Me, 5-OMe-indole-3-acetic acid is probably caused by its specific conformational preferences which preclude optimal interactions between the carboxyl group and the hydroxyl groups of β-cyclodextrin. The shorter retention time of the indole-3-acrylic acid, on the other hand, can be attributed to the reduced flexibility of its 3-side chain. Non-acidic derivatives, unlike the indole carboxylic acids, are poorly retained on the column. The observed correlation between the affinities of the indole derivatives for ABP1 and their retention factors seems to indicate that in general the indole-3-carboxylic acids have higher hydrogen bonding and zinc ion complexing capacity than the non-acidic indole derivatives. On the other hand, the observed parallelism between relative binding affinities of indole-3-acetic acids for HSA and their retention times is mostly result of the fact that the binding cavities of both HSA and β-cyclodextrin are lipophilic in nature and that they are also large enough to accommodate these compounds.
